# One-year outcomes of an innovative laparoscopic pectopexy procedure using inverted T-mesh for treatment of advanced uterine and anterior vaginal prolapse

**DOI:** 10.1038/s41598-026-40730-0

**Published:** 2026-02-26

**Authors:** Evelyn Yang, Ching-Pei Tsai, Pao-Sheng Shen, Tsung-Ho Ying, Tsung-Hsien Lee, Gin-Den Chen, Yun-Han Liao, Man-Jung Hung

**Affiliations:** 1 Department of Obstetrics and Gynecology, Chung Shan Medical University Hospital, No. 110, Section 1, Jianguo N Rd, South District, Taichung City, Taiwan, ROC; 2 Department of Obstetrics and Gynecology, School of Medicine, College of Medicine, Chung Shan Medical University, No. 110, Section 1, Jianguo N Rd, South District, Taichung City, Taiwan, ROC; 3 Department of Obstetrics and Gynecology, Taichung Veterans General Hospital, No. 1650, Section 4, Taiwan Boulevard, Taichung, Taiwan, ROC; 4 Department of Statistics, Tunghai University, No. 1727, Section 4, Taiwan Boulevard, Taichung City, Taiwan, ROC

**Keywords:** Pelvic organ prolapse, Laparoscopy, Pectopexy, Sacrocolpopexy, Levator avulsion, Diseases, Medical research

## Abstract

This prospective pilot study was conducted to evaluate the safety and efficacy of an innovative laparoscopic pectopexy (LP) technique employing an inverted T-mesh for the concomitant treatment of advanced uterine and anterior vaginal prolapse. 42 patients (62.7%) underwent the innovative LP procedure, while 25 patients (37.3%) with predominant uterine or vaginal vault prolapse underwent conventional laparoscopic sacral hysteropexy or colpopexy (LS). The primary outcome was the anatomic outcomes and functional results. Secondary outcomes included surgical complications and reoperations. At baseline, the demographics of the LP group was younger, with lower body mass index (BMI), and were more sexually active when compared to the LS group (*P* < 0.05). At one-year follow-up, the anatomic cure rate (POP-Q stage < 2) was significantly lower in the LP group (78.6% [33/42] vs. 92.0% [23/25]; *P* = 0.0108). Functional outcomes were assessed using the PFDI-20, POPIQ-7 and PISQ-12 questionnaires, and significant improvements were observed in all domains. Multiple logistic regression revealed that the type of surgical procedure was not a significant predictor of outcome. Levator avulsion was the only variable significantly associated with poorer results (*P* < 0.05). Both groups demonstrated favorable postoperative outcomes, and had no severe surgical complications. Limitations of this study included non-randomization of the sample groups, small sample size and a follow-up time of one year. Despite these limitations, the findings suggest that the innovative LP procedure is a safe and feasible option for the concomitant management of advanced uterine and anterior vaginal prolapse. However, levator ani avulsion markedly increases the risk of anatomic recurrence. This study was registered on ClinicalTrials.gov (NCT07411898; 13/02/2026).

**Supplementary Information:**

The online version contains supplementary material available at 10.1038/s41598-026-40730-0.

## Introduction

Apical suspension is a cornerstone of pelvic organ prolapse (POP) surgery, with sacrocolpopexy widely regarded as the gold standard due to its high cure rate and durability^1–3^. Recently, laparoscopic sacrocolpopexy has gained popularity, and laparoscopic sacral hysteropexy is also a feasible alternative for women seeking uterine preservation. However, both procedures are technically demanding, associated with steep learning curves and prolonged operating times^4,5^. Additionally, these surgeries are challenging in patients with extensive intra-abdominal adhesions and carry risks such as ileus, bowel injury, and bowel obstruction^6–8^. Further recent improvements in minimally invasive apical suspension techniques and the growing field of natural orifice based apical repairs have also broadened the scope of prolapse repair and have shown promising results with less invasive transvaginal routes^9,10^.

Since its introduction in 2011, laparoscopic pectopexy, which employs bilateral mesh fixation to the bilateral iliopectineal ligament, has been proposed as an effective alternative for apical prolapse repair^11^. Previous studies have found similar results in anatomical and functional outcomes from both procedures, while pectopexy had fewer post-operative bowel complications and lateral-defect cystocele^12–14^. Moreover, pectopexy has a more advantageous learning curve and shorter operating times^5,12,15^. Despite these advantages, the majority of existing studies are retrospective in design, limited by small sample sizes or lack of control groups^5,11,12^.

Patients referred for pelvic reconstructive surgery frequently present with combined anterior and apical prolapse. Previous studies found that anterior compartment involvement is the most common and serious defect that occurs with an apical defect^16^. To address this, many surgeons will conduct concomitant surgeries in addition to sacrocolpopexy or pectopexy. This prospective pilot study was conducted before a planned multicenter randomized controlled trial to explore the surgical outcomes and safety of an innovative laparoscopic uterine pectopexy technique using inverted T-meshes for simultaneous apical and anterior vaginal repair. This study hypothesized that the addition of an anterior extension of the mesh during a laparoscopic pectopexy was a safe and effective procedure while also providing concomitant repair of anterior and uterine compartment prolapse, while utilizing laparoscopic sacrocolpopexy procedures conducted during the same period as a reference for efficacy and safety.

## Materials and methods

### Study design

This was a prospective pilot study conducted at a tertiary hospital. Between August 2020 and December 2023, patients who underwent laparoscopic reconstructive surgery for advanced POP were consecutively enrolled. In brief, the inclusion and exclusion criteria were presentation with a symptomatic uterine prolapse ≧ POP-Q stage 2 in patients who wished to retain the uterus, no known uterine or cervical pathology, no previous prolapse mesh repair, and no diseases known to affect bladder or bowel function. The selection of surgical procedures was based on a pre-operative split-speculum vaginal examination to assess the anterior, middle, and posterior vaginal compartments separately. If patients had a concomitant advanced uterine and apical compartment prolapse, they were counseled regarding native tissue repair and mesh repair. For patients who chose mesh repair, transvaginal and transabdominal mesh repair were discussed. However, for younger patients, we suggested transabdominal mesh repair and both the sacrocolpopexy and pectopexy procedures were discussed, and appropriate counseling was conducted. An innovative laparoscopic uterine pectopexy (LP) procedure using inverted T-shaped mesh was performed in patients presenting with both advanced uterine and anterior vaginal prolapse. Patients with predominant uterine (or vaginal vault) prolapse without obvious anterior vaginal prolapse underwent the conventional laparoscopic sacral hysteropexy/colpopexy (LS) procedure. Before any procedures were performed, all patients provided informed consent, and the research was conducted in accordance with the Declaration of Helsinki. The primary outcome measures were anatomic outcomes and functional results. Anatomic outcomes, including prolapse cure rates, were assessed by evaluating the POP-Q stages and parameters. Functional results were assessed using the PFDI-20, POPIQ-7 and PISQ-12 questionnaires. Secondary outcomes included surgical complications and reoperations. This study was performed in accordance with standard guidelines and was approved by the Chung Shan Medical University Hospital institutional ethics committee (CSMUH No:CS2-21047). This study was registered on ClinicalTrials.gov (NCT07411898).

### Baseline assessment

All patients underwent a pelvic examination including a cough stress test, a multichannel urodynamic study with prolapse reduction, and a structured interview utilizing composite condition-specific questionnaires including Mandarin versions of the Pelvic Floor Distress Inventory (PFDI-20), Pelvic Organ Prolapse Impact Questionnaire-7 (POPIQ-7) and the short-form Pelvic Organ Prolapse/Urinary Incontinence Sexual Questionnaire (PISQ-12)^17,18^. Prolapse severity and pelvic floor muscle assessment was assessed using the Pelvic Organ Prolapse Quantification system (POP-Q) and the PERFECT scheme^19^. All urodynamic studies and urodynamic definitions followed the definitions and standards proposed by the International Urogynecological Association and the International Continence Society^20^.

### Surgical intervention

**Fig. 1:**
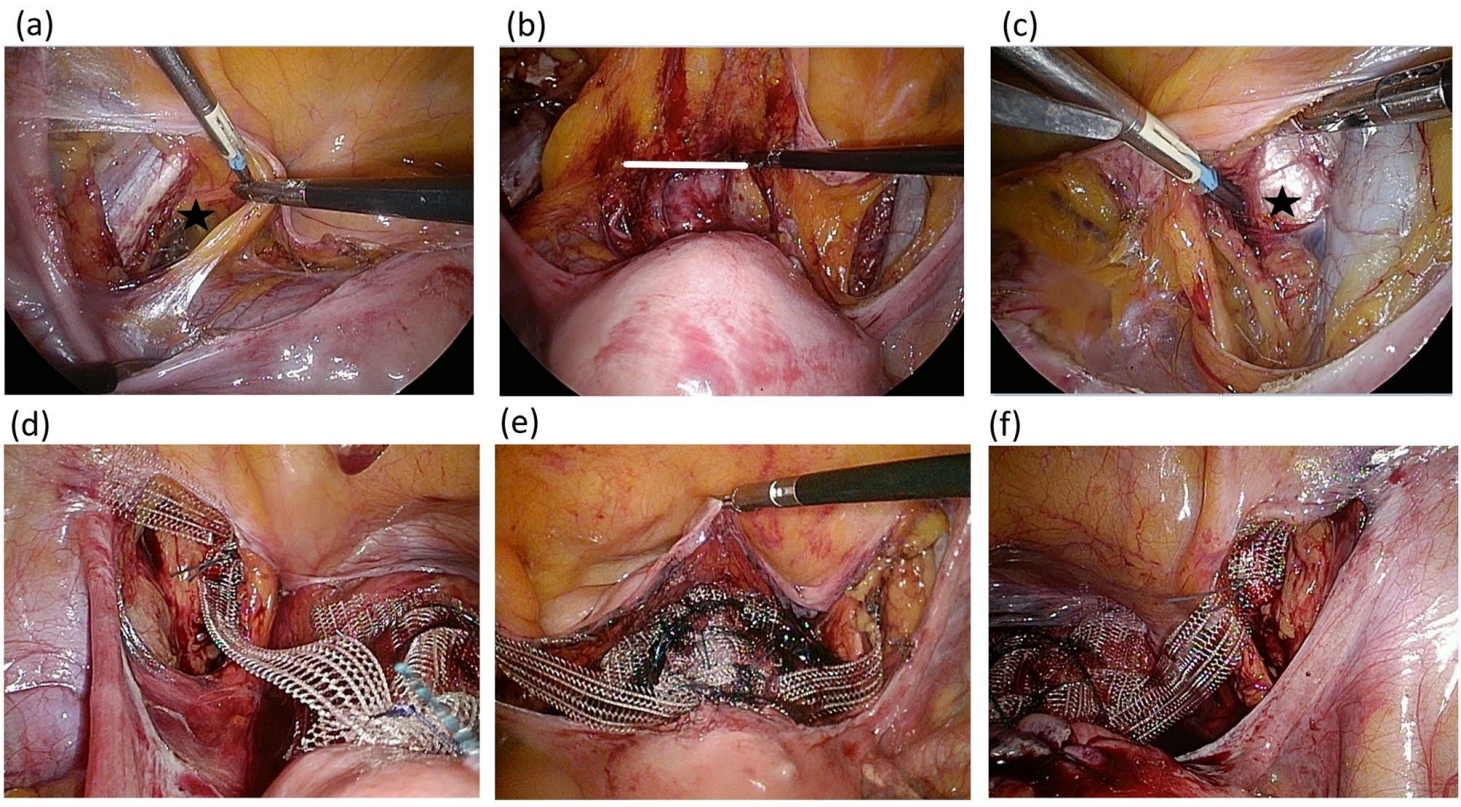
Steps of the laparoscopic pectopexy with inverted T-mesh operation. The peritoneum was opened along the pubic bone between the left round ligament and the left medical umbilical ligament to expose the iliopectineal ligament (*black star*) (**a**). The anterior peritoneum of the uterus and bladder was dissected distally to the level of the bladder neck (*white line*) (**b**). The right iliopectineal ligament (*black star*) was exposed (**c**). The uterus was then placed in its natural position without tension, the central portion of the mesh was sutured to the anterior cervix and the endopelvic fascia of the anterior vaginal wall with 1-O V-Loc sutures in a continuous clockwise fashion (**e**), and the two lateral arms of the mesh were fixed to the bilateral iliopectineal ligaments using interrupted 2-O Ethibond sutures (**d**, **f**).

**Fig. 2:**
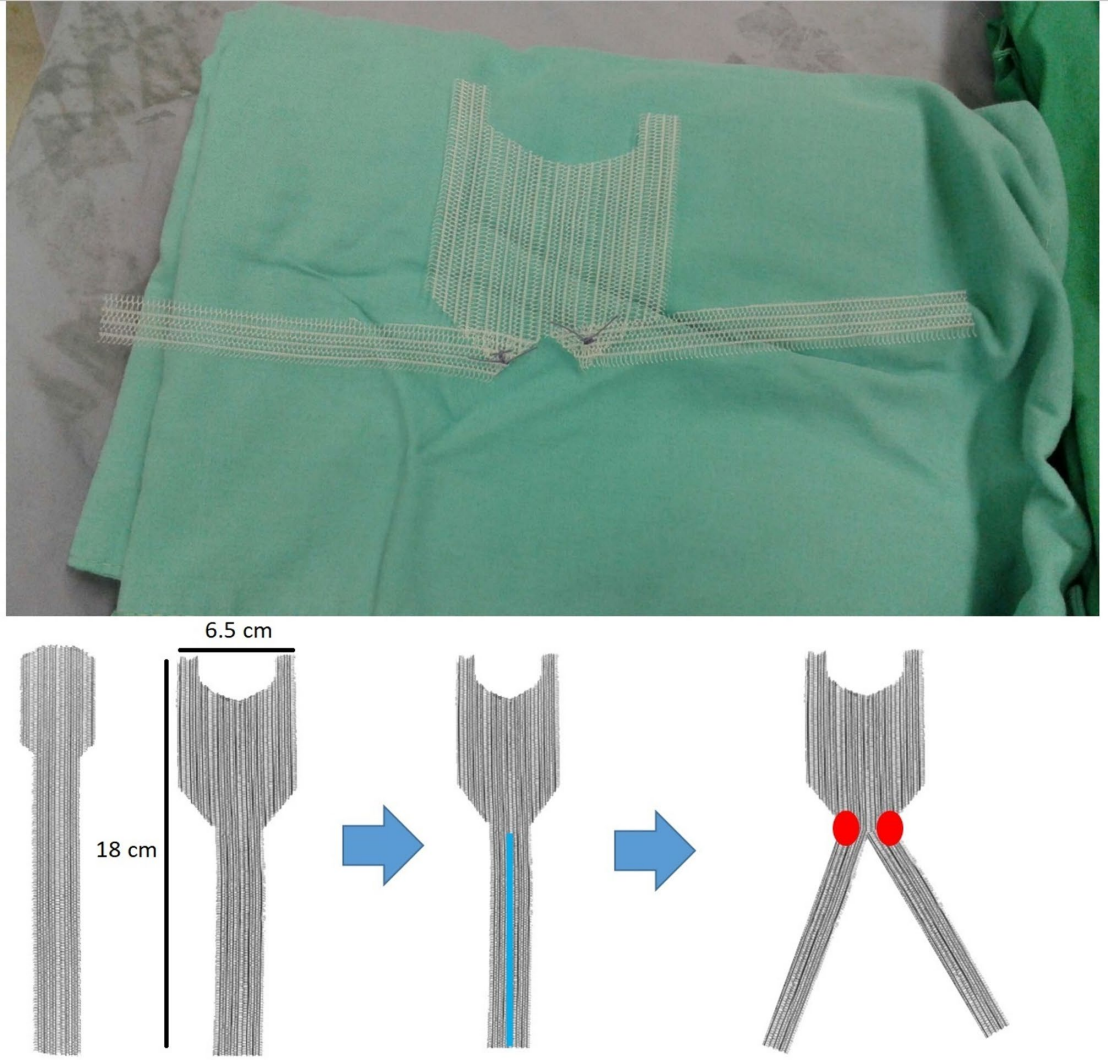
An inverted T-shaped mesh was made by utilizing the anterior piece of the PELVI-STOP mesh (6.5 cm × 18 cm) and cutting the tail into two halves. Then, two sutures were placed at the intersection of the tail and the main piece of mesh to form an inverted T shape.

The LS procedure was performed as previously published^21,22^. The steps of the innovative LP procedure are illustrated in Fig. 1. A uterine manipulator was first used to position the uterus in retroversion. The anterior peritoneum of the uterus and bladder was dissected distally to the level of the bladder neck and laterally to the pelvic sidewalls at the iliopectineal ligament. An inverted macroporous polypropylene monofilament T-shaped mesh was fashioned from the anterior mesh of an industry-designed, commercialized double-mesh (PELVI-STOP, APIS Technologies, Switzerland) for sacral suspension as in Fig. 2. The central portion of the mesh was sutured to the anterior cervix and the endopelvic fascia of the anterior vaginal wall with 1-O V-Loc sutures (Covidien, Mansfield, MA, USA) in a continuous clockwise fashion with at least six fixation points and deeper anchoring of the mesh at the cervix. The uterus was then placed in its natural position without tension, and the two lateral arms of the mesh were fixed to the bilateral iliopectineal ligaments using interrupted 2-O Ethibond sutures (Ethicon, Somerville, NJ, USA) with at least two fixation points at each arm to ensure adequate attachment of the mesh. The peritoneum was closed continuously. If clinically indicated, concomitant surgeries were conducted. However, no patients underwent a concomitant hysterectomy. All procedures were performed by the same urogynecologist, with over 20 years of experience. Cystoscopy was performed prior to the conclusion of surgery. Postoperatively, all patients underwent transurethral bladder drainage, and a voiding trial was initiated on postoperative day 3. The voiding trial was successful if the patient was able to void freely and the post-void residual was < 25% of the total bladder volume and < 100 mL on two separate occasions.

### Follow-up investigation

Perioperative data were obtained from medical records. Operation time and estimated blood loss included all concomitant procedures. Follow-up examinations were conducted at the outpatient clinic at 1 week, 6 weeks, 3 months, 12 months, and annually thereafter. At each visit, pelvic examinations were performed to assess POP-Q stages, incontinence, pelvic floor conditions, levator ani avulsion and any adverse events. Vaginal digital palpation was conducted to assess pelvic muscle strength and levator ani avulsion. Anatomical recurrence was defined as POP-Q stage ≥ 2 in any compartment within one year. Functional outcomes were assessed by comparing pre- and post-operative questionnaire scores. Dyspareunia was defined as a score of ≥ 2 to question 5 of the PISQ-12 questionnaire at the 12-month follow-up.

### Statistical analysis

Clinical data are presented as mean ± SD, median (range) or percentage when appropriate. Univariate analysis was used to compare the demographic and various parameters between groups. The association between anatomic outcomes and important clinical variables was assessed by multivariate logistic regression analyses. To prevent overfitting and unstable estimates, additional LASSO (Least Absolute Shrinkage and Selection Operator) processes for logistic regression with Akaike Information Criterion (AIC) were performed. The Goodness-of-Fit (*P*-values = 0.9889, 0.8147, 0.733 and 0.819, respectively) of the multiple logistic models tested with the method described by Hosmer and Lemeshow indicated that the models fit very well. A *p*-value of < 0.05 was considered statistically significant. Statistical analyses were performed using SAS 9.2 software (SAS Institute Inc, Cary, NC).

## Results

### Patient characteristics

A total of 67 women were included in this study. LP was performed on 42 (62.7%) patients. The remaining 25 (37.3%) patients, who had either dominant uterine prolapse (N = 11) or post-hysterectomy vaginal vault prolapse (N = 14), underwent conventional laparoscopic sacral hysteropexy/colpopexy (LS). Patient demographics are presented in Table 1. Preoperatively, patients in the LP group were younger, had a lower BMI and were more sexually active. Pre-operative urodynamic diagnoses were similar in both groups.

**Table 1:** Preoperative characteristics of patients who underwent laparoscopic pectopexy (LP) or laparoscopic sacrocolpopexy or sacrohysteropexy (LS).

Patient characteristics	LP (N = 42)		LS (N = 25)		*P* value
	Value	Range	Value	Range	
General data					
Mean age (year)	56.6 ± 12.2	(36–85)	65.4 ± 13.7	(42–91)	0.0085^a^
Mean parity	2.2 ± 0.8	(0–4)	2.6 ± 0.87	(2–4)	0.1358^a^
Mean body mass index (kg/m^2^)	23.3 ± 2.9	(16.2–28.55)	25.5 ± 2.97	(18.7–38.7)	0.0233^a^
% Menopause	52.4%	(22/42)	72.0%	(18/25)	0.4000^b^
% Sexual activity	50.0%	(21/42)	16.0%	(4/25)	0.0054^b^
% Diabetes mellitus	4.76%	(2/42)	8.0%	(2/25)	0.5885^b^
% Hypertension	19.0%	(8/42)	32.0%	(8/25)	0.2291^b^
Urodynamic diagnoses					
% Bladder hypersensitivity	38.1%	(16/42)	40.0%	(10/25)	0.8770^b^
% Detrusor overactivity	11.9%	(5/42)	0.0%	(0/25)	0.0729^b^
% Urodynamic stress incontinence	42.86%	(18/42)	44.0%	(11/25)	0.9272^b^
% Bladder outlet obstruction	23.81%	(10/42)	32.0%	(8/25)	0.4645^b^
% Detrusor underactivity	2.38%	(1/42)	12.0%	(3/25)	0.1080^b^

^a^Kruskal-Wallis *p*-value; ^b^Chi-Square *p*-value.

### Surgical results

Surgical outcomes are presented in Table 2. Perioperative data were comparable between groups. Concomitant surgeries were performed on an individualized basis. The incidences of post-operative complications were clinically acceptable and comparable between groups. The most common complication was de novo urgency urinary incontinence, observed in 9.5% of LP patients and 8.0% of LS patients (*P* = 0.976). No severe adverse events were reported. However, a statistically significant difference was observed in prolapse recurrence, defined as POP-Q stage ≥ 2 in any compartment at 1-year follow-up. The anatomic cure rate was significantly lower in the LP group compared to the LS group (78.57% (33/42) vs. 92.0% (23/25); *P* = 0.0108).

**Table 2:** Surgical results of patients who underwent laparoscopic pectopexy (LP) or laparoscopic sacrocolpopexy or sacrohysteropexy (LS).

	LP (N = 42)		LS (N = 25)		
Parameters	Value	Range	Value	Rage	P value
Peri-operative data					
Mean hospital stay (days)	5.19 ± 1.02	(4–10)	5.92 ± 1.99	(4–12)	0.1337^a^
Mean Foley drainage (days)	3.79 ± 0.84	(3–8)	3.8 ± 1.32	(2–8)	0.7380^a^
Mean operating time (minutes)	187.14 ± 47.35	(120–315)	191.68 ± 40.47	(120 ~ 255)	0.4518^a^
Mean estimated blood loss (ml)	65 ± 56.68	(5–200)	67.8 ± 75.06	(10–250)	0.7316^a^
Mean pain score (next day)	2.38 ± 1.411	(0–6)	2.64 ± 1.24	(0–6)	0.3330^a^
Concomitant surgeries					
% Trachelectomy	19.0%	(8/42)	20.0%	(5/25)	0.9240^b^
% McCall Culdoplasty	95.2%	(40/42)	0.0%	(0/25)	0.0001^b^
% Mid-urethral sling	42.86%	(18/42)	51.2%	(11/25)	0.7773^b^
% Ant/Post Colporrhaphy	47.62%	(20/42)	48.0%	(12/25)	0.8264^b^
% Myomectomy	16.70%	(7/42)	0.0%	(0/25)	0.0310^b^
% Adhesiolysis	9.50%	(4/42)	4.0%	(1/25)	0.4053^b^
% Salpingoophorectomy	2.40%	(1/42)	0.0%	(0/25)	0.4370^b^
Surgical effectiveness in 1 year					
% Pelvic organ prolapse (≦ stage 1)	78.57%	(33/42)	92.0%	(23/25)	0.0108^b^
% Stress urinary incontinence (cure)	100.0%	(18/18)	100.0%	(11/11)	0.1930^b^
Surgical complications					
% Perioperative blood transfusion	0.0%	(0/42)	0.0%	(0/25)	
% Pelvic hematoma formation	0.0%	(0/42)	0.0%	(0/25)	
% Pelvic inflammatory disease	0.0%	(0/42)	0.0%	(0/25)	
% Delayed free voiding (> 7 days)	2.38%	(1/42)	4.0%	(1/25)	0.7064^b^
% De novo stress incontinence	0.0%	(0/42)	0.0%	(0/25)	
% De novo urgency incontinence	9.52%	(4/42)	8.0%	(2/25)	0.9760^b^
% Vaginal mesh extrusion	2.38%	(1/42)	0.0%	(0/25)	0.4370^b^
% Trocar wound infection	0.00%	(0/42)	8.0%	(2/25)	0.0627^b^

^a^. Kruskal-Wallis *p*-value; ^b^. Chi-Square *p*-value

Anatomic outcomes, assessed by comparing the POP-Q stages and parameters, are shown in Table 3. Post-operative pelvic assessments at one-year follow-up were unable to be conducted for one patient in the LP group and 5 patients in the LS group due to inability to return to the clinic. However, all patients completed one-year follow-up questionnaires. Both procedures resulted in significant improvement across all stages with similar anatomic cure rates (POP-Q stage < 2) in the anterior (90.2% vs 93.0%), middle (80.5% vs 95.0%), and posterior (95.1% vs. 100.0%) compartments (*P* > 0.05). Notably, in contrast to the lower success rate (80.5%) in the middle compartment, LP achieved high cure rates (90.2%) in the anterior compartment (i.e., with a low rate of residual cystocele) and significantly improved Aa and Ba points (*P* < 0.001). Significant improvement was also noted in all individual POP-Q parameters, except for total vaginal length (TVL), which remained stable after LP (from 8.67 ± 0.95 to 8.77 ± 0.89 cm; P = 0.23). In addition to the apical suspension procedures, the performance of concomitant surgeries could also contribute to the changes of various POP-Q parameters (e.g. GH, PB, etc.) postoperatively. No significant between-group differences were found in post-operative POP-Q stages or individual parameters.

**Table 3:** Anatomic outcome assessed by Pelvic Organ Prolapse Quantification (POPQ) system in patients who underwent laparoscopic pectopexy (LP) or laparoscopic sacrocolpopexy or sacrohysteropexy (LS) at one-year follow-up.

	LP with t-mesh					LS					*P* value post-op groups
	Pre-op (N = 42)		Post-op (N = 41)		*P* value	Pre-op (N = 25)		Post-op (N = 20)		*P* value	
POPQ stages											
Anterior site					< .0001^a^					< .0001^a^	0.99^c^
Stage 0-I	1	(2.38%)	37	(90.24%)		2	(8.0%)	18	(93.0%)		
Stage II	24	(57.14%)	2	(4.88%)		6	(24.0%)	2	(6.9%)		
Stage III	11	(26.19%)	2	(4.88%)		4	(16.0%)	0	(0.0%)		
Stage IV	6	(14.29%)	0	(0.0%)		13	(52.0%)	0	(0.0%)		
Apical site					< .0001^a^					< .0001^a^	0.15^c^
Stage 0-I	1	(2.38%)	33	(80.49%)		1	(4.0%)	19	(95.0%)		
Stage II	20	(47.62%)	8	(19.51%)		3	(12.0%)	1	(5.0%)		
Stage III	14	(33.33%)	0	(0.0%)		6	(24.0%)	0	(0.0%)		
Stage IV	7	(16.67%)	0	(0.0%)		15	(60.0%)	0	(0.0%)		
Posterior site					< .0001^a^					< .0001^a^	0.34^c^
Stage 0-I	12	(28.57%)	39	(95.12%)		6	(24.0%)	20	(100.0%)		
Stage II	17	(40.48%)	2	(4.88%)		6	(24.0%)	0	(0.0%)		
Stage III	8	(19.05%)	0	(0.0%)		4	(16.0%)	0	(0%)		
Stage IV	5	(11.90%)	0	(0.0%)		9	(36.0%)	0	(0%)		
POPQ parameters											
Aa	1.07 ± 1.22		− 2.24 ± 1.14		< .0001^b^	1.88 ± 1.56		− 2.4 ± 0.94		< .0001^b^	0.58^c^
Ba	1.68 ± 1.90		− 2.26 ± 1.28		< .0001^b^	4.16 ± 3.25		− 2.35 ± 0.93		< .0001^b^	0.94^c^
C	2.17 ± 2.95		− 4.99 ± 2.89		< .0001^b^	5.44 ± 3.46		− 6.3 ± 2.27		< .0001^b^	0.10^c^
Ap	0.14 ± 1.96		− 2.39 ± 0.83		< .0001^b^	0.92 ± 2.19		− 2.75 ± 0.44		< .0001^b^	0.07^c^
Bp	0.48 ± 2.41		− 2.37 ± 0.92		< .0001^b^	2.8 ± 3.84		− 2.7 ± 0.47		< .0001^b^	0.14^c^
D	− 1.88 ± 4.43		− 7.19 ± 3.47		< .0001^b^	− 0.12 ± 4.90		− 8.65 ± 1.22		< .0001^b^	0.07^c^
GH	3.42 ± 1.12		2.98 ± 0.76		0.03^b^	3.06 ± 0.99		3.23 ± 0.77		0.04^b^	0.17^c^
PB	2.54 ± 0.74		3.1 ± 0.71		< .001^b^	2.38 ± 0.82		3.18 ± 0.73		< .0001^b^	0.72^c^
TVL	8.67 ± 0.95		8.77 ± 0.89		0.23^b^	8.38 ± 1.21		8.78 ± 1.04		< .0001^b^	0.76^c^

^a^Wilcoxon signed-rank test; ^b^Paired-t test; ^c^Wilcoxon rank-sum test.

Functional outcomes were assessed using the PFDI-20, POPIQ-7 and PISQ-12 questionnaires, evaluating symptoms, quality of life and sexual function (Table 4). Significant improvements were observed in all domains, with the exception of four (16%) sexually active patients in the LS group who reported stable sexual function scores (32.75 ± 6.34 to 34.75 ± 7.41; *P* = 0.25). No cases of de novo dyspareunia were reported in either group. Furthermore, a significant decrease in the incidence of dyspareunia was noted in the LP group (from 57 to 24%; *P* < 0.002).

**Table 4:** Functional outcome assessed by comparing the pre- and post-operative symptoms and quality-of-life scores in patients who underwent laparoscopic pectopexy (LP) or laparoscopic sacrocolpopexy or sacrohysteropexy (LS) at one-year follow-up.

	LP (N = 42)					LS (N = 25)					*P* value postop groups
	Pre-op values		Post-op values		*P* value	Pre-op values		Post-op values		*P* value	
PFDI-20	21.98 ± 7.80		4.52 ± 5.94		< .0001^a^	24 ± 9.01		3.4 ± 3.82		< .0001^a^	0.82^b^
POPDI-6	10.95 ± 4.32		1.33 ± 3.64		< .0001^a^	11.24 ± 4.19		0.2 ± 0.5		< .0001^a^	0.08^b^
UDI-6	7.93 ± 4.56		1.83 ± 2.75		< .0001^a^	9.08 ± 4.99		1.88 ± 2.19		< .0001^a^	0.56^b^
CRADI-8	2.98 ± 3.5		1.26 ± 2.35		< .0001^a^	2.88 ± 3.05		1.56 ± 2.47		< .001^a^	0.36^b^
POPIQ-7	12.17 ± 4.49		2.55 ± 2.87		< .0001^a^	14.24 ± 4.74		1.56 ± 2.47		< .0001^a^	0.44^b^
PISQ-12	31.71 ± 6.29		37.67 ± 6.05		< .0001^a^	32.75 ± 6.34		34.75 ± 7.41		0.25^a^	0.58^b^
Dyspareunia	57.0%	(12/21)	24.0%	(5/21)	< 0.04^ac^	0%	(0/4)	0%	(0/4)		

^a^Wilcoxon test; ^b^Mann–Whitney test; ^c^McNemar test.

### Outcome associations

Table 5 shows the results of univariate analyses comparing clinical variables between patients with and without recurrence. Further multivariate logistical regression analyses identified levator avulsion (− 2.312) as the only significant covariate associated with poor surgical outcomes (Supplementary Table 1). Levator avulsion was associated with an odds ratio of 8 (*P* = 0.0116; 95% confidence interval 1.36–47.02). Notably, levator avulsion was detected in 7 (16%) patients in the LP group. The anatomic recurrence rates were significantly higher in patients with levator avulsion, who underwent LP, than those without levator avulsion (57.1% (4/7) vs. 14.3% (5/35)). In addition to levator avulsion, heavy lifting was associated with worse surgical outcomes after LP with a marginally statistical significance (Supplementary Table 2). Further LASSO regression with AIC disclosed results consistent with the multivariate logistic regression analyses. Of the covariates investigated (Supplementary Table 1, 2), levator avulsion was found to be the only significant variable that has close association with poor anatomic outcomes (*P* = 0.0431) in this study and prolapse recurrence after LP (*P* = 0.0427), respectively.

**Table 5:** Comparisons of important clinical variables after laparoscopic pelvic organ prolapse repair to determine possible risk factors for surgical failure.

	Cure (n = 56)		Failure (n = 11)		
	Value	Range	Value	Range	*P* value
Surgical Intervention LP with t-mesh LS	33 (58.9%) 23 (41.1%)		9 (81.8%) 2 (18.2%)		0.1513^a^
Patient characteristics					
Mean age (years)	60.6 ± 14.2	36–91	56.5 ± 8.2	42–71	0.3971^b^
Mean body mass index (kg/m^2^)	24.2 ± 3.2	16.2–32.0	23.7 ± 5.2	20.2–38.7	0.1024^b^
Mean parity	2.4 ± 0.9	0–5	2.3 ± 0.8	1–4	0.6332^b^
Previous prolapse surgery	19.6%	11/56	0.0%	0/11	0.1079^a^
Hypertension	21.4%	12/56	36.4%	4/11	0.2882^a^
Diabetes mellitus	5.4%	3/56	9.1%	1/11	0.6328^a^
Chronic cough	0.0%	0/56	0.0%	0/11	
Occupation requires heavy lifting	12.5%	7/56	18.2%	2/11	0.6134^a^
Chronic constipation	16.1%	9/56	36.4%	4/11	0.1197^a^
Levator ani avulsion	5.4%	3/56	36.4%	4/11	0.0021^a^
Pre-operative POP-Q parameters					
Ba	2.6 ± 2.9	− 2 to + 7	2.7 ± 2.2	− 2 to + 7	0.6726^b^
C	3.4 ± 3.7	− 5 to + 8	3.2 ± 2.9	− 1 to + 8	0.9589^b^
Bp	1.4 ± 3.3	− 2 to + 7	1.3 ± 2.9	− 2 to + 7	0.8974^b^
Pre-operative POP-Q stages					0.5915^a^
I	1.8%	1/56	0.0%	0/11	
II	33.9%	19/56	36.4%	4/11	
III	28.6%	16/56	45.5%	5/11	
IV	35.7%	20/56	18.2%	2/11	

^a^Chi-square *p*-value; ^b^Kruskal-Wallis *p*-value.

Table 6 showcases the individual characteristics of the 11 (16.4%) patients who had prolapse recurrence. Of the two patients who presented with anatomic recurrence in the LS group, one patient had undergone a laparoscopic sacral hysteropexy and had a recurrent uterine and anterior vaginal prolapse. The other patient had undergone a laparoscopic sacrocolpopexy and had a recurrent anterior vaginal prolapse. Of the nine patients with recurrence in the LP group, two patients had recurrent anterior vaginal prolapse and underwent transvaginal mesh procedure and seven patients had recurrent stage 2 (Point C − 1 to + 0.5) uterine prolapse and underwent a Manchester operation. A Manchester operation was conducted for recurrent moderate uterine prolapse, with or without cervical elongation, because of its proven history of better composite outcomes of success when compared with the sacrospinous hysteropexy in uterus preserving prolapse surgery^23^. Of the two patients with recurrent anterior vaginal prolapse, one patient exhibited levator ani avulsion and one patient had an occupation that required heavy lifting.

**Table 6:** Characteristics of the 11 patients who had anatomic recurrence (POPQ stages ≧ 2) at one year follow-up.

Case	Age	Operation	Potential risk factors	POPQ parameters Pre-/Post-operative			Subsequent management
				Ba	C	Bp	
1	42	Laparoscopic sacrohysteropexy	Constipation	+ 3/0	+ 4/0	− 2/− 3	Manchester operation was recommended at 1 year follow-up but the patient preferred observation
2	59	Laparoscopic sacrocolpopexy	Nil	+ 7/0	+ 8/− 7	+ 7/− 3	No symptoms → Observation
3	49	LP with t-mesh	Heavy lifting	+ 3/+ 1	+ 4/+ 1.5	+ 2/+ 1	Uterine and anterior vaginal wall suspension with vaginal mesh (MIPS) and cervix amputation at 9 months post-LP. Subsequent follow-up at 1 year showed no prolapse.
4	67	LP with t-mesh	Levator avulsion	+ 5/+ 2.5	+ 8/− 5	+ 5/− 2	Anterior vaginal wall suspension with vaginal mesh (MIPS) at 6 months. Subsequent follow-up at 1 year showed no prolapse.
5	59	LP with t-mesh	Nil	+ 1/− 3	+ 1/0	+ 1/− 3	Manchester operation was performed at 1 year follow-up
6	54	LP with t-mesh	Nil	− 1/− 2	− 1/0	− 1/− 2	Manchester operation due to cervical elongation at 7 months. Subsequent follow-up at 1 year showed no prolapse.
7	71	LP with t-mesh	Heavy lifting	+ 3/+ 1	+ 4/0	+ 2/+ 1	Manchester and McCall culdoplasty at 14 months
8	58	LP with t-mesh	Levator avulsion	+ 4.5/+ 0.5	+ 2.5/+ 0.5	+ 2/− 2	Manchester and anterior colporrhaphy with Kelly plication at 13 months
9	59	LP with t-mesh	Constipation	+ 2/− 2	+ 2.5/+ 0	+ 2/− 2	Manchester operation due to cervical elongation at 7 months post-LP
10	49	LP with t-mesh	Constipation and levator avulsion	+ 1/− 2	+ 1/0	− 2/− 2	Manchester operation due to cervical elongation at 5 months post-LP
11	54	LP with t-mesh	Constipation and levator avulsion	+ 1/− 2	+ 1/− 1	− 2/− 1	Manchester operation at 12 months post-LP

## Discussion

This study introduces an innovative operative procedure utilizing an inverted T-mesh during laparoscopic pectopexy to concomitantly repair advanced stages of anterior and apical vaginal prolapse. Since it is a concomitant repair, a single piece of mesh is utilized to cover the anterior vaginal wall while also performing a lateral suspension to the bilateral iliopectineal ligaments. Furthermore, this operation is able to preserve the uterus while restoring the natural axis of the pelvic floor.

The major differences between the two groups were mean age, mean BMI, and the percentage of women who were sexually active. The results suggested that, after clinical counselling and individualized treatment plans, younger, more sexually active women tended to favor the LP procedure; whereas older, less sexually active patients and patients with previous prolapse repair more often opted for the LS procedure. Only two (4.8%) patients in the LP group had undergone previous prolapse repair which showed that the majority of the LP group was a primary prolapse repair and not due to a failure of previous operations. 14 patients presented with vaginal vault prolapse and underwent the LS operation.

In this study, the innovative LP with inverted T-mesh had favorable and promising perioperative outcomes. Previous studies found shorter operation times, lower blood loss and shorter hospitalization stays for the LP procedure when compared to LS^5,8,12,24,25^. In a systematic review, Parsaei et.al., found statistically significant shorter operation times for LP when compared to LS and lower estimated blood loss^24^. In this study, no significant differences were found in hospitalization days, operating time, and estimated blood loss between both groups. These findings were likely due to the surgeries which were performed concomitantly on a case-by-case basis. The operation time and estimated blood loss included all the surgeries conducted during the operation. Furthermore, 16.7% (7/42) patients in the LP group underwent a concomitant myomectomy while 95.2% (40/42) of the LP group underwent a prophylactic concomitant McCall culdoplasty to prevent future posterior vaginal compartment prolapse. The concomitant McCall culdoplasty was for enterocele prevention and was conducted by bilateral uterosacral ligament plication to narrow the widened cul-de-sac and differed from a high McCall which was similar to a colpopexy procedure.

At 1-year follow-up, the POP-Q stages and individual parameters showed similar outcomes between both groups. However, the objective anatomical cure rate was significantly different with the LP group having a 78.8% (33/42) cure rate and the LS with a 92.0% (23/25) cure rate (*P* = 0.0108). Of the nine patients with recurrence in the LP group, seven patients had recurrent stage 2 uterine prolapse and underwent a subsequent Manchester operation to address the recurrence. Previous studies have found comparable outcomes between LS and LP when investigating apical compartment prolapse but these studies have mostly investigated milder prolapse stages without uterus preservation or studies did not analyze uterine preservation as a separate group^5,11,12,24,26^. The difference in cure rate in this study was likely due to uterus preservation in all LP patients as well as the advanced stages and multiple compartments of prolapse included. In contrast to LS patients, patients who underwent LP were on average younger and tended to have larger uterine volumes. The weight of the preserved uterus may have contributed to a higher recurrence rate by exerting downward traction on the apical support, similar to the effect of a bilateral suspension acting like a swing. Furthermore, the downward traction could be more exaggerated in LP patient with levator avulsion due to weakened pelvic support.

In contrast to the lower success rate (80.5%) in the middle compartment, LP achieved high cure rates (90.2%) in the anterior compartment (i.e., cystocele repair). Before the placement of the fashioned T-mesh, a wide dissection was made at the vesico-vaginal space distally to the level of the bladder neck and laterally to the pelvic sidewalls. The central portion of the T-mesh was then sutured to the anterior cervix and the endopelvic fascia for a full coverage of the anterior vaginal wall. The two lateral arms of the T-mesh were fixed to the bilateral iliopectineal ligaments. We suggest the way the T-mesh was anchored should contribute to an augmented anterior vaginal support like a hammock^27^.

Favorable functional outcomes were achieved after both procedures. Symptoms and quality of life scores all improved significantly which were similar to findings in previous studies^8,28,29^. While sexual function scores improved in the LS group, this improvement did not reach significance. This is likely due to the low percentage of sexually active women in this group. In contrast, improved sexual function scores and decreased incidence of dyspareunia were found in the sexually active patients after LP.

Few studies have analyzed possible risk factors for recurrence. Sato et al. previously found advanced preoperative stage and adhesiolysis as independent risk factors^30^. However, few studies have included characteristics such as levator ani avulsion, chronic constipation or chronic heavy lifting. In this study, in both univariate and multivariate analyses, levator avulsion was identified as a strong risk factor for surgical failure. Vaginal digital palpation was conducted to assess pelvic muscle strength and to educate the patient regarding proper pelvic floor muscle training. Upon visual inspection and palpation of levator ani avulsion, oftentimes a large genital hiatus is observed and there is no contractile tissue palpated on the pubic ramus^31^. While sonographic studies were not conducted, Dietz et al. found that vaginal examination by an experienced physician was noninferior in diagnosing levator avulsion injury but it is possible to miss more minor levator ani injuries^31^. In the eleven recurrent cases, four presented with levator avulsion. The prevalence of levator avulsion was 16% in the LP group and 0% in the LS group. If the patients with levator ani avulsion were excluded in the LP group, the anatomic cure rate was 82.5% which was comparable to the 90% cure rate of LS. Levator avulsion is strongly associated with pelvic organ prolapse and weaker pelvic muscles which increase the risk of recurrence^32^. Therefore, levator ani muscle strength should be integrated in routine pre-operative pelvic exams and studies to assess for the increased risk of prolapse repair failure. In patients with identified pre-operative levator ani muscle avulsion, careful counseling and pre-operative surveys should be conducted.

No severe complications were noted in this study. De novo urgency urinary incontinence was the most prevalent complication after both procedures with a 9.52% (4/42) rate in LP and 8.0% (2/25) in LS which are similar to previous studies^8^. This complication could potentially be due to some irritative effects of the implanted meshes due to the extensive bladder dissection. All cases of de novo urgency urinary incontinence were mild and controllable by oral medications.

The limitations of this study were the non-randomization of the patient population which resulted in some differences between groups at baseline, relatively small sample size and short follow-up time of one year. This was a prospective study without randomization between the two patient populations which analyzed the feasibility of the inverted T-mesh during laparoscopic pectopexy for concomitant uterine and anterior vaginal prolapse suspension. All patients were counseled pre-operatively by the same surgeon and counseling was based on the findings on the split speculum exam. This study shows only the short-term follow-up results at one year post-operation. Further long-term analyses are needed to assess the durability of the inverted T-mesh. The strengths were a homogenous patient population and multiple logistic regression analyses to avoid potential confounding factors as well as identify possible predisposing factors of surgical failure.

## Conclusion

Our pilot study results suggest the innovative LP procedure using inverted T-mesh is a safe procedure for a concomitant repair of advanced uterine and anterior vaginal prolapse and results in favorable anatomical and functional outcomes at one-year post-operation. By repeated statistical analyses, we found that levator avulsion is a significant predisposing factor that affects recurrence after LP. The information obtained in this study should be helpful for patients, who are referred and counselled for a reconstructive pelvic surgery, in shared decision making and surgical planning.

## Supplementary Information

Below is the link to the electronic supplementary material.

Supplementary Material 1

Supplementary Material 2

## Data Availability

The datasets generated during and/or analysed during the current study are available from the corresponding author on reasonable request.
