# Supplementary material for: One-year outcomes of an innovative laparoscopic pectopexy procedure using inverted T-mesh for treatment of advanced uterine and anterior vaginal prolapse
Source: Sci Rep. 2026 Feb 26;16:11202. doi: 10.1038/s41598-026-40730-0 (PMC13046823; doi:10.1038/s41598-026-40730-0)
Supplement: Supplementary file 1 — Supplementary Material 1 [file 41598_2026_40730_MOESM1_ESM.docx]

Supplementary Table 2. Multivariate logistical regression analysis to identify possible risk factors associated with poor surgical outcomes after laparoscopic uterine pectopexy (N=42).

| **Analysis of Maximum Likelihood Estimates** | | | | | |
| --- | --- | --- | --- | --- | --- |
| **Parameter** | **DF** | **Estimate** | **Standard Error** | **Wald Chi-Square** | **Pr > ChiSq** |
| Intercept | 1 | -7.8747 | 11.4757 | 0.4709 | 0.4926 |
| Age | 1 | -0.0404 | 0.0641 | 0.3963 | 0.5290 |
| BMI | 1 | 0.3605 | 0.3162 | 1.3000 | 0.2542 |
| Constipation | 1 | -1.8812 | 1.5939 | 1.3929 | 0.2379 |
| Levator Avulsion | 1 | 4.2006 | 1.9791 | 4.5049 | 0.0338 |
| Heavy Lifting | 1 | -4.5330 | 2.5161 | 3.2456 | 0.0716 |
| Ba (Pre-OP) | 1 | 0.5203 | 0.9900 | 0.2762 | 0.5992 |
| C (Pre-OP) | 1 | 0.5027 | 0.9555 | 0.2768 | 0.5988 |
| Bp (Pre-OP) | 1 | -1.2462 | 1.1602 | 1.1538 | 0.2828 |
| Anterior Prolapse Stage (Pre-OP) | 1 | -2.8196 | 2.6731 | 1.1126 | 0.2915 |
| Apical Prolapse Stage (Pre-OP) | 1 | 3.7393 | 4.3945 | 0.7240 | 0.3948 |
| Posterior Prolapse Stage (Pre-OP) | 1 | -0.0289 | 2.0097 | 0.0002 | 0.9885 |
