# Supplementary material for: One-year outcomes of an innovative laparoscopic pectopexy procedure using inverted T-mesh for treatment of advanced uterine and anterior vaginal prolapse
Source: Sci Rep. 2026 Feb 26;16:11202. doi: 10.1038/s41598-026-40730-0 (PMC13046823; doi:10.1038/s41598-026-40730-0)
Supplement: Supplementary file 2 — Supplementary Material 2 [file 41598_2026_40730_MOESM2_ESM.docx]

Supplementary Table 1. Multivariate logistical regression analysis to identify possible risk factors associated with poor surgical outcomes (N=67).

| **Analysis of Maximum Likelihood Estimates** | | | | | |
| --- | --- | --- | --- | --- | --- |
| **Parameter** | **DF** | **Estimate** | **Standard Error** | **Wald Chi-Square** | **Pr > ChiSq** |
| Intercept | 1 | -1.4470 | 7.8363 | 0.0341 | 0.8535 |
| Operation Type | 1 | -2.4586 | 1.9211 | 1.6378 | 0.2006 |
| Age | 1 | 0.0171 | 0.0438 | 0.1521 | 0.6965 |
| BMI | 1 | -0.1455 | 0.1590 | 0.8376 | 0.3601 |
| Constipation | 1 | -2.8579 | 1.8104 | 2.4921 | 0.1144 |
| Levator Avulsion | 1 | -2.9698 | 1.4236 | 4.3520 | 0.0370 |
| Heavy Lifting | 1 | -1.4723 | 1.2354 | 1.4204 | 0.2333 |
| Ba (Pre-OP) | 1 | 0.3566 | 0.7028 | 0.2575 | 0.6118 |
| C (Pre-OP) | 1 | -0.4611 | 0.6463 | 0.5089 | 0.4756 |
| Bp (Pre-OP) | 1 | -0.7676 | 0.6862 | 1.2514 | 0.2633 |
| Anterior Prolapse Stage (Pre-OP) | 1 | -1.3343 | 1.7687 | 0.5692 | 0.4506 |
| Apical Prolapse Stage (Pre-OP) | 1 | 2.5116 | 2.4063 | 1.0894 | 0.2966 |
| Posterior Prolapse Stage (Pre-OP) | 1 | 1.4641 | 1.5882 | 0.8489 | 0.3566 |
